# Nonfunctional distractor analysis: An indicator for quality of Multiple choice questions

**DOI:** 10.12669/pjms.36.5.2439

**Published:** 2020

**Authors:** Madiha Sajjad, Samina Iltaf, Rehan Ahmed Khan

**Affiliations:** 1 Dr. Madiha Sajjad, FCPS. Department of Pathology, Riphah International University, Rawalpindi, Pakistan; 2 Dr. Samina Iltaf, MPhil. Department of Pathology, Riphah International University, Rawalpindi, Pakistan; 3 Dr. Rehan Ahmed Khan, FCPS, FRCS, JM-HPE, MSc HPE, PhD. Department of Surgery, Riphah International University, Rawalpindi, Pakistan

**Keywords:** Non-functional distractor, Distractor efficiency, Multiple choice question, Item writing flaws

## Abstract

**Objectives:**

To analyze the low to medium distractor efficiency items in a multiple-choice question (MCQ) paper for item writing flaws.

**Methods:**

This qualitative study was conducted at Islamic International Medical College Rawalpindi, in October 2019. Archived item- analysis report from a midyear medium stakes MCQ paper of 2^nd^ year MBBS class, was analyzed to determine the non-functional distractors (NFDs) and distractor efficiency (DE) of items, in a total of 181 MCQs. DE was categorized as low (3-4 NFDs), medium (1-2 NFDs) and high (0 NFD). Subsequently, qualitative document analysis of the MCQ paper whose item analysis report was assessed was conducted to investigate the item flaws in the low to medium DE items. The flaws identified were coded and grouped as, within option flaws, alignment flaws between options and stem/ lead-in and other flaws.

**Results:**

Distractor efficiency was high in 69 items (38%), moderate in 75 items (42%) and low in 37 items (20%). The item-writing flaws identified in low to moderate DE items within distractors included, non-homogenous length (1.8%), non-homogenous content (8%) and repeat in distractor (1.7%). Alignment flaws between distractors and stem/ lead-in identified were linguistic cues (10%), logic cues (12.5%) and irrelevant distractors (16%). Flaws unrelated to distractors were low cognitive level items (40%) and unnecessarily complicated stems (11.6%).

**Conclusions:**

Analyzing the low to medium DE items for item writing flaws, provides valuable information about item writing errors which negatively impact the distractor efficiency.

## INTRODUCTION

Post hoc item analysis is a commonly used tool to assess the quality of Multiple-choice questions (MCQ) based examinations in undergraduate medical education. It provides useful information about the reliability and validity of test items. The parameters commonly assessed in item analysis are; the discrimination index (DI), difficulty index and distractor efficiency (DE). Functional or efficient distractors are those, chosen by more than 5% of examinees whereas the distractors chosen by less than 5% examinees are known as non-functional distractors.^1^ For distractors to be effective they should all be plausible and if possible, none should be incorrect.^2^ The number of non-functional distractors NFDs in an MCQ item determines the distractor efficiency of that item. Designing plausible distractors and reducing the number of non-functional distractors (NFDs) improves the quality of the test.

In an MCQ item, the context or background is referred to as the ‘stem’, followed by the question known as the ‘lead-in’ and a number of option choices. High-quality MCQs require a well written unambiguous stem, clear lead-in and rational choice of options. In a one best type of MCQ, one of the options is the correct response known as the ‘key’ while others are described as ‘distractors’.^3^ Item writing flaws in MCQs which are not related to item construct occur when there is a breach in following the standard item-writing guidelines with reference to language and grammatical structure, style of writing the stem and option choices.^4^ Various types of item writing flaws are found in literature, for example; Long correct answer, logic cues, grammar cues, ‘except’ or ‘not’ in the lead-in, inconsistent language in options etc.^5^ Some types of Item flaws cue the student to the correct answer, assisting students who are ‘test wise’. ‘Test-wiseness’ refers to students’ ability to recognize the answer in MCQs without employing their content-related reasoning skills or knowledge. Other types of flaws may mislead the students towards selecting wrong options.^4^ This may over or under-estimate student performance, thus introducing a source of error that negatively effects the validity of student scores.^4^,^6^

In depth study of items showing low distractor efficiency can help test-developers and instructors understand test-wiseness of students in relation to item flaws. Distractor efficiency also has an indirect effect on the item difficulty as well as its discriminatory ability.^7^ Removing the non-functional distractors from MCQ items in some cases, restores the distractor efficiency of item to optimal level. In other cases, identifying distractor related flaws and correcting them can improve distractor efficiency and positively impact the item difficulty and discrimination indices.^8^

Not much work has been done on the qualitative aspect of individual items with low distractor efficiency. The objective of this study was to analyze the item writing flaws in low to medium distractor efficiency items in a multiple-choice question (MCQ) paper in order to gain insight into the structural flaws in items which negatively impact the distractor efficiency, overall exam quality and student performance. This study will help the test developers be aware of item flaws and address them in a more logical and systematic manner.

## METHODS

This qualitative study was conducted at Islamic International Medical College, Riphah International University in October 2019. Ethical approval was taken from Institutional review committee of Islamic International Medical college, Ref #Riphah/IIMC/IRC/20/005.

Archived item- analysis data report from a midyear medium stakes MCQ paper of 2^nd^ year MBBS class was analyzed to assess the distractor efficiency of items. There were 181 single best response type MCQs having a reliability coefficient of 0.88. One hundred and six (106) items had five option choices and 75 items had four option choices.

Nonfunctional distractors (NFD) were identified as the distractors chosen by less than 5% examinees. Distractor efficiency (DE) was defined on the basis of the number of NFDs in an item and ranged from 0-100%. Distractor efficiency of items was graded as low (having 3-4 NFDs), medium (having 1-2 NFDs) and high (having 0 NFD).

Subsequently, qualitative document analysis of the MCQ paper whose item analysis report was assessed, was carried out independently by two reviewers, who evaluated each low to moderate DE item, for item writing flaws. The reviewers were experienced in MCQ test item development and were also trained in MCQ writing. Item flaws were investigated with reference to item writing guidelines proposed by Haladyna et al. and followed in literature.^9^-^11^ After consensus, the flaws identified were coded and grouped as:

- Flaws within options
- Alignment flaws between options and stem/ lead-in
- Other flaws, unrelated to options or their alignment with stem/ lead-in

## RESULTS

A total of 649 distractors were identified in 181 MCQs out of which 205 were nonfunctional distractors (31.6%). Out of these 181 MCQ items, 112 items were low to moderate distractor efficiency items. Distractor efficiency of items was determined as given in Table-I.

**Table-I:** Distractor efficiency of items.

No of Non-functional distractors (NFD’s)		Distractor efficiency	No. of items
*4 option items*	*5 option items*		
0	0	High (100%)	69 items (38%)
1-2	1-2	Moderate (50-75%)	75 items (42%)
3	3-4	Low (< 50%)	37 items (20%)

Out of the 112 low to moderator distractor efficient items, 62 items (34.2% of all MCQ items) had one or more item writing flaws in the option choices or their alignment with the stem and lead-in. Of the remaining 50 items; flaws unrelated to the options or their alignment with stem/ lead-in were found in 27 items, these were low cognition level items or complicated stems; whereas no item writing flaw was identified in 23 items. The item flaws identified are given in Table-II.

**Table-II:** Frequency of item writing flaws in low and moderate distractor efficiency items.

Within options flaws (11.5%)		Alignment flaws between options and item stem/ lead in (38.5%)		Other flaws	
Distractor non-homogenous in length	1.8% (3)	Linguistic cues	10% (11)	Low cognitive level items	40% (45)
Distractor non-homogenous in content	8% (9)	Logic cues	12.5% (14)	Unnecessarily complicated/ unfocused stem	11.6% (13)
Distractor repeated in same item	1.7% (2)	Limited possible or irrelevant distractors	16% (18)	No item writing flaw	20.5% (23)

The most frequently identified flaws were ‘low cognitive level items’ (40%), ‘irrelevant/ limited possible options or distractors’ (16%), followed by ‘logic cues’ and (12.5%) and, unnecessarily complicated stems (11.6%).

## DISCUSSION

We aimed to identify different types of qualitative item flaws in MCQ items having low to moderate distractor efficiency. Distractor efficiency was low to moderate in 62% items in our study. Various studies show comparable results with low to moderate distractor efficient items in the range of 31% to 75% in various local studies,^1^,^12^,^13^ and 50-86% in international studies.^6^,^14^

The frequency of flawed items in the 112 low to moderate distractor efficient items in our study was 89/112, (79%) which was 49% of the total MCQ items. In a study by Pais et al., 55.8% items had at least one item writing flaw. This high frequency was similar to other studies, where around half of the items contained item writing flaws.^5^,^6^,^11^,^15^,^16^ Flaws in items included limited plausible distractors, clues, unfocused stems, errors in writing option choices or those related to cognitive level chosen etc. These flaws may cue the students and cause the distractors to be chosen by students based on their ‘guessing’ skills rather than content-specific cognitive skills.^11^,^17^

Lower-order cognition items were a frequently identified flaw (40%). Ideally constructed MCQs should be written at a level of difficulty appropriate to level of the students and the focus of assessment should not be students’ knowledge of inconsequential or trivial facts.^18^ The low cognition item stems based on recall are sometimes too easy for the level/ grade of students, causing students to not consider any distractor as an option. In a study by Testa et al, items categorized at the ‘Application level’ were more distractor efficient when compared to items labelled at ‘Knowledge’ and ‘Comprehension’ levels.^19^ In multiple studies low cognition items were in the range of 40-60%., as was the case in our study^11^,^20^

The predominant item flaw identified following the ‘low cognition items’ was ‘limited number of possible logical or plausible distractors’ (16%) which was in the category Alignment flaws between the distractors and the stem or lead-in’. In a study by Salih et al., implausible distractors were cited as a frequent flaw (25%).^20^ Pham et al. observed that students who can ‘rule out’ options based on their ability to assess their plausibility can narrow down their guessing to fewer options than the five which are frequently employed in MCQs.^4^ Test developers are generally asked to provide four or five option choices and it may be difficult for them to furnish quality distractors every time, as some questions inherently have less plausible options. According to studies by and Tarrant et al. and Haladyna et al., the quality of distractors rather than their number is the crucial requisite and suggested the minimum required number of options in an item should be considered in the context of the ability to develop plausible distractors even reducing the number of options to just three.^6^,^9^

Another frequently identified flaw in our study was ‘logic cues’ due to inadvertent use of specific words or themes in distractors and stem or lead-in directing towards the correct answer (12.5%). In some other studies these were less frequent (4-6%).^5^,^11^

Ambiguous/ confusing stem or lead-in was found to be 11.6% in our study. In various studies the frequency of this flaw ranged from 19-50%.^5^,^11^,^20^ Vague or ambiguous terms effect the ability of students to answer a question correctly.^18^ One of the reasons for these flaws in our study may be that English is not the primary language in the study setting, although it is the medium of instruction (EMI).^6^ The impact of language on the psychometric properties, due to item quality as well as the examinees’ comprehension needs to be further studied.

The least frequent flaws in our study were in the category, ‘within options flaws’ (11.5%). This was in accordance with the study by Salih et al.^20^ However other studies show a higher frequency of these flaws.^11^ The reason for a lower frequency of ‘within option flaws’ in our study may be, because of a system of pre-hoc analysis for medium and high stakes examination items at our institution. This may lead to reduction in such obvious flaws in item writing as, ‘all of the above’ or ‘none of the above’ options etc.^20^Also, having a faculty development program in place to train faculty in standard item writing guidelines also helps improve the quality of MCQs.^11^

No item writing flaw was identified in 20% items having low to moderate distractor efficiency. One of the possible reasons may be that the item psychometric statistics from only one administration of the test was studied, and the options identified as non-functioning distractors in this exam may have been subject to sampling bias and may perform quite differently in other samples.^6^

### Limitation of the study

A limitation of the study is, that a single paper was analyzed for item flaws based on its item analysis index of low DE.

## CONCLUSION

Analyzing the low to medium DE items for item writing flaws, provides valuable information about item writing errors which negatively impact the distractor efficiency. Correcting the errors can improve distractor efficiency and overall exam quality.

### Authors’ Contribution

**RAK:** Conceived, designed reviewed and did final approval.

**MS and SI:** Did data collection, manuscript writing, editing and review.

All authors are responsible and accountable for the accuracy and integrity of the work.
